# Improvement Path for Resource-Constrained Cities Identified Using an Environmental Co-Governance Assessment Framework Based on BWM-mV Model

**DOI:** 10.3390/ijerph18094969

**Published:** 2021-05-07

**Authors:** Jian Wang, Jin-Chun Huang, Shan-Lin Huang, Gwo-Hshiung Tzeng, Ting Zhu

**Affiliations:** 1 School of Business, Quzhou University, Kecheng District, Quzhou 324000, China; 16010042@pxu.edu.cn (J.W.); 16010043@pxu.edu.cn (J.-C.H.); 2 E-Commerce Research Center, Pingxiang University, Anyuan District, Pingxiang 337055, China; 3 Department of Tourism Management, College of Economics and Management, Sanming University, Sanyuan District, Sanming 365004, China; shadow0518713@gmail.com; 4 National Park Center, Sanming University, Sanyuan District, Sanming 365004, China; 5 Graduate Institute of Urban Planning, College of Public Affairs, National Taipei University, San Shia District, New Taipei 23741, Taiwan; ghtzeng@gm.ntpu.edu.tw

**Keywords:** environmental governance, collaboration of stakeholders, BWM-mV model, environmental co-governance system, local governments with limited resources, budget or time

## Abstract

Global warming and extreme weather have increased most people’s awareness of the problem of environmental destruction. In the domain of sustainable development, environmental governance has received considerable scholarly attention. However, protecting and improving the environment requires not only substantial capital investment but also cooperation among stakeholders. Therefore, based on the network structure of stakeholders, the best–worst method (BWM) and modified Vlsekriterijumska Optimizacija I Kompromisno Resenje method were combined to form an environmental co-governance assessment framework that can be used to evaluate the effects of various policies and identify strategies for further improvement through data analysis (henceforth the BWM-mV model). This mechanism is not only useful for evaluating the effectiveness of environmental governance policies but also for generating suggestions to enhance these policies. Hence, the BWM-mV model is particularly suitable for local governments with limited resources in time, money, or labor. Pingxiang City Government is currently subject to such limitations and was therefore selected as the subject of an empirical case study. The results of this study revealed that the aspects (i.e., criteria) the Pingxiang City Government should urgently improve on pertain to a high-quality information communication platform (*C*_13_) and smooth joint decision-making by stakeholders (*C*_24_).

## 1. Introduction

Urban environmental pollution is a problem that has caused widespread concern in recent years [1]. Industrial agglomeration is a primary contributor to urbanization [2], and intense industrial development has caused many environmental problems in cities [3], such as increased energy consumption [4], carbon emissions [5,6], and air pollution [7]. These problems pose numerous risks to public health [8]. The environmental problems caused by urbanization and industrialization urgently require solutions [9,10]. Bennett and Satterfield [11] suggested that in many environmental protection initiatives, the main approach to solving environmental problems should be the establishment of a strong local environmental governance system, and Cheng and Li [12] discussed the impact of environmental governance policies on the industrial environment.

The government plays the role of the manager in an environmental governance system. To ensure sustainable human activity in natural environments, the government enacts various management policies and regulations. However, although these policies and regulations can achieve ecological protection, they also affect the development of the local economy and, in turn, the lives of residents. This one-way governance model usually faces certain challenges when implemented. For example, companies producing polluting products may choose to move to other cities with weaker environmental supervision rather than consider adopting an approach to co-existing with the local environment [13]. Lockwood et al. [14] advocated that a good governance model establishes a cooperative relationship between governments and citizens in public affairs. Scholars have further emphasized that this cooperative management relationship should not be limited to the relationship between governments and citizens but should involve all stakeholders and encourage these stakeholders to participate more actively in public affairs and government decision-making [15,16]. To cope with the complexity of the environment and the uncertainty of the future, a highly adaptable system that requires stakeholders to conduct adequate exchanges must be established for environmental governance. Through complete exchanges of information, stakeholders can seek out other stakeholders who share common interests to actively promote environmental protection [17,18,19]. Thus, forming a joint governance model that involves a nation’s government, enterprises, and citizens is crucial for protecting the environment and promoting environmental sustainability [20].

“Evaluation” not only benefits environmental governance but also has a significant impact on environmental policy development, management processes, and improvements in decision-making quality [21,22]. Cheng and Li [12] posited that environmental governance assessment is the highest priority among tasks that promote a city’s sustainable development. In this context, Bennett and Satterfield [11] developed an environmental governance assessment framework and analytic method. This framework has four goals, namely that a policy be “effective”, “equitable”, “responsive”, and “robust”, and these goals include 19 indicators. Gao et al. [22] combined the observation periods of three groups (governments, enterprises, and the public) to analyze their interests and then proposed an environmental governance evaluation framework. The framework has contributed to environmental governance systems, but it pays more attention to the relationship among the interests of a nation’s government, enterprises, and public in environmental governance and less to how co-governance can be conducted. Moreover, governance processes should be based on the concepts of transparency, participation, and shared accountability [23,24]. These are not only the main attributes of environmental governance but also the legal rights of stakeholders [25]. Therefore, this paper proposes that environmental governance must be a process in which stakeholders participate, share responsibilities, and demonstrate a willingness to assume relevant responsibilities when realizing common interests through full information disclosure. Additionally, this paper introduces an evaluation indicator framework called the environmental co-governance assessment framework (ECAF).

Urban environmental protection is a systematic and complex project [1] that often requires substantial economic investment [26]. The development of the urban economy restricts the level of environmental governance [27]. As the executors of environmental governance, local governments are also naturally restricted by their economic level [28,29,30]. Therefore, cities lacking resources (i.e., resource-constrained cities) must accurately formulate improvement strategies when conducting environmental governance to make more effective use of limited resources. More research is necessary to explore the methods of environmental co-governance in local government when resources are heterogeneous. Therefore, the purpose of this study is to develop a method, which can be applied to actual cases, for providing improvement suggestions for resource-poor cities from the perspective of environmental co-governance.

The BWM-mV model is a type of multiple-criteria decision-making (MCDM) methodology [31] that includes two components. The first is the best–worst method (BWM), which can be used to determine the importance of each criterion. Decision makers can determine the key to achieving a goal by referring to the weight of each criterion [32,33,34,35]. The second component is the modified Vlsekriterijumska Optimizacija I Kompromisno Resenje (VIKOR), an approach for determining the distance between each criterion and the best state (aspiration level), which is referred to as the gap [36,37]. Decision makers can identify room for improvement in each criterion according to its gap [38]. Scholars formerly used this model to evaluate alternative criteria, but rarely used it for improving the alternatives [39,40,41,42]. However, the most important feature of the modified VIKOR is that it can reveal whether room for improvement remains for each criterion [43,44]. Thus, this model should be used for not only the evaluation but also the improvement of alternative criteria. Moreover, an advantage of the BWM is that it can be used to quickly obtain the criteria importance ranking with a certain degree of accuracy [45]. Decision-makers can reexamine the rationality of resource use according to the importance and gap of each criterion. This means the BWM-mV model can effectively provide suggestions for improvements to decision makers who are under time and financial constraints.

Pingxiang City is a prefecture-level city in western Jiangxi Province, China. An abundance of coal resources enabled this city to achieve modern industrial development relatively early. However, since 2007, the coal resources of Pingxiang have been in a period of exhaustion, and in 2008, the city was listed as one of the first resource-exhausted cities in China. Given the problems faced by resource-based traditional industries, the economic development of Pingxiang has also been greatly affected. The fiscal deficit is increasing year by year. To redevelop the local economy, the government of Pingxiang transferred high-pollution industries to the east. However, this development model has caused a crisis in the urban environment, and decision-makers in China are aware that these crises will have devastating consequences. Therefore, every local government must give thought to environmental governance, and problems concerning environmental governance in Pingxiang urgently require solutions. Therefore, Pingxiang was selected in this study as a resource-constrained city to demonstrate the operation of an environmental governance system based on the Environment Co-governance Assessment Framework (ECAF).

This study had three objectives: (1) construct an assessment framework for environmental co-governance called the ECAF; (2) use the BWM-mV model to construct an analytical mechanism for environmental co-governance that enables decision-makers to more clearly grasp how aspects for improvement ought to be prioritized when resources are limited; and (3) help cities with limited resources, such as Pingxiang City, to obtain suggestions on how environmental governance can be improved. The remainder of this paper is arranged as follows. The second section details the development of a preliminary indicator framework for environmental co-governance through discussions of the relevance of stakeholders and environmental governance. In addition, a preliminary indicator framework for constructing the ECAF is examined. Subsequently, the method for using the BWM-mV model in environmental co-governance is explained, and the operating process of the model is described. The third section discusses the environmental governance of Pingxiang City through a data analysis and offers suggestions for improvement. Finally, the principal findings of this paper are summarized.

## 2. Environmental Co-Governance Framework and Process of BWM-mV Model

**Figure 1:**
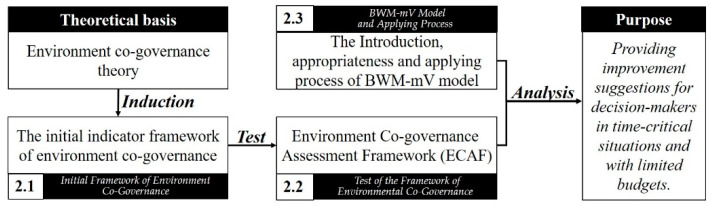
Planning and purpose of this section.

To construct a framework for environmental co-governance, this study proposes an initial indicator framework that combines factors affecting environmental governance obtained from the literatures. Additionally, a test was conducted to ensure the effectiveness of the indicator framework, which consequently determined the effectiveness of the environmental co-governance framework. At the end of this section, an explanation of the process of applying the BWM-mV model is provided. This section comprises three subsections: Section 2.1 introduces the initial indicator framework of environment co-governance, Section 2.2 summarizes the test conducted on this framework, and Section 2.3 details how the BWM-mV model is applied. The contents of these subsections are outlined in Figure 1.

### 2.1. Initial Framework of Environment Co-Governance

Many scholars have proposed theories on governance issues. Most of these theories distinguish among government, corporate, and public stakeholders and posit that the relationship among those stakeholders can be classified into three structural types [46]. The first is the top-down type, in which the government is perceived as the initiator of problem-solving projects. Private firms and the public mainly adhere to government requirements. Because this type is government-led, the results of planning and policy are consistent, and a plan can be implemented relatively efficiently [47,48]. However, this type is sometimes prone to the problem of inconsistency between the planning direction and the needs of the public [49]. The second type is the bottom-up type [50]. This type is mostly based on initiation by private firms or the public, and the government usually plays the roles of collaborator and supervisor. This model can solve the problem of inconsistency between a planning direction and the needs of the people [51]. This type is mainly employed to encourage people to state their goals and then make plans accordingly, and the government and private firms only play supporting roles. This type is more efficient in practice, mainly due to sufficient communication and public support during the planning period, which can effectively reduce the amount of time required for future project implementation [52]. The third type is the network type, which has no fixed initiator. With full information disclosure, stakeholders can fulfill “common interests” through communication and collaboration [53,54]. The process includes the main concepts of transparency, participation, and joint decision-making and responsibility [17,18,19].

An increasing amount of research has provided evidence suggestion that environmental problems cannot be solved by a single organization alone and that cooperation may be the more feasible approach [55]. Therefore, the aim of environmental governance should be to effectively achieve the common interest in environmental protection. Under information transparency, stakeholders participate in governance actions and share responsibilities. Additionally, some evidence suggests that mutual trust among stakeholders can help achieve the goal of environmental protection [56,57,58]. Delgado-Márquez et al. [59] contended that trust is the foundation of stakeholder partnerships. Unlike the general environmental governance model, the environmental co-governance system is based on mutual trust among stakeholders. The approach we advocate is a pluralistic, free, open, and flexible model. The model is based on trust and is goal oriented, allowing stakeholders to conveniently, voluntarily, and proactively provide corrective information and participate in environmental governance. Through the pursuit of common interests, it also achieves environmental protection [60,61,62]. Accordingly, the environmental governance framework developed in this study is based on three dimensions, namely the correctness and fluidity of public information (*D*_1_), effectiveness of and engagement in environmental co-governance actions (*D*_2_), and the effect and binding force of environmental governance mechanisms (*D*_3_).

Information acquisition is the basis for stakeholders to jointly address environmental matters [63,64]. Information transparency refers to information sharing among stakeholders [9,65]. For example, the government and citizens can ascertain the status of environmental pollution through environmental responsibility reports disclosed by private firms [66]. For this reason, establishing a mechanism of “restraint, reward, and care” is crucial for fostering an environment of mutual trust and willingness to communicate and encourage stakeholders to proactively provide relevant information [67,68,69,70]. In addition, because the information comes from different stakeholders, checking, summarizing, and re-organizing the information is also critical for ensuring that the information is correct and complete [71]. The technical purpose of an information exchange platform is to allow stakeholders to exchange information, and the platform’s operation performance is a critical factor affecting information quality [72]. Therefore, the correctness and fluidity of public information (*D*_1_) refers to the proactive provision of relevant information in a timely and convenient manner based on mutual trust among stakeholders. This information must be correct, reliable, and complete. This dimension has three criteria, namely an atmosphere conducive to the proactive provision of information (*C*_11_), correct and complete information (*C*_12_), and a high-quality information communication platform (*C*_13_).

Stakeholder participation can improve the level and effectiveness of environmental governance [9,25]. Effective environmental governance actions ensure the effectiveness of environmental governance policies and mechanisms while also increasing the attention and participation of all stakeholders [22,73]. First, although government policies reflect directions of environmental protection development, allowing for the adjustment and refinement of policies to ensure their suitability in local contexts is key under constantly changing environmental and social conditions [11,74]. Encouraging companies and members of the public to propose relevant plans from the perspective of user needs is an appropriate approach for making such adjustments [75]. Thus, for the formation of a diverse environmental governance mechanism, the initiators of governance actions should be diverse; they can be a government entity, companies, or members of the public. Second, because environmental joint governance actions should adopt a goal-oriented approach and simultaneously use multiple governance mechanisms, confirming whether an implemented project can achieve the goal of protecting or improving the ecological environment is critical [11,76]. Third, to ensure the effectiveness of multiple environmental governance mechanisms and environmental protection projects, a sound supervision mechanism must be established [30,77]. This includes the confirmation of supervisors, the designation of their responsibilities, and the formulation of supervisory policies and regulations, supervisory methods, and improvement measures. Finally, stakeholder participation in the decision-making process is a crucial aspect of environmental co-governance. To ensure that joint decision-making by stakeholder’s progresses smoothly, an environment must be established in which stakeholders can easily communicate and coordinate with each other [30,78]. In general, the effectiveness of environmental co-governance actions has a major influence on whether environmental protection goals are achieved. This dimension has four criteria, namely diversified environmental governance mechanisms (*C*_21_), effective environmental protection projects (*C*_22_), robust co-management and monitoring mechanisms (*C*_23_), and smooth joint decision-making by stakeholders (*C*_24_).

**Figure 2:**
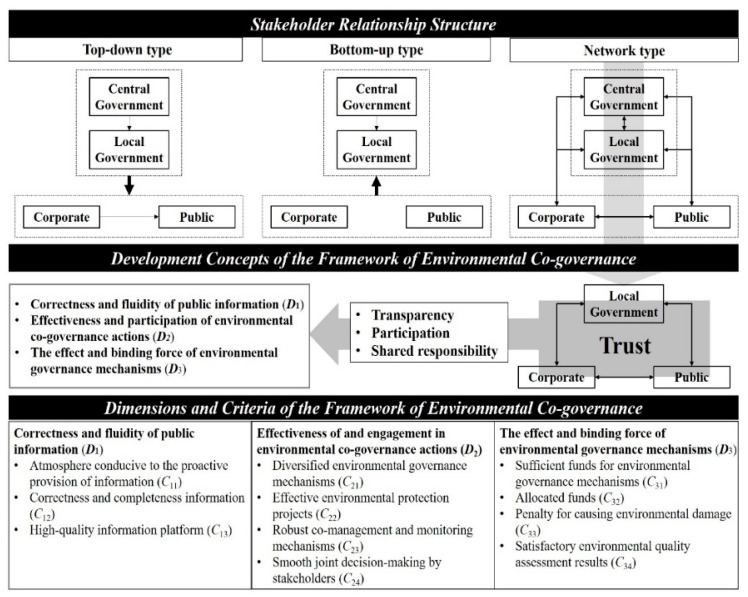
Concept and framework of environmental co-governance.

Because the environmental co-governance system has no clear initiator, a responsibility constraint mechanism based on the concept of the pursuit of common interests is required to ensure the successful completion of environmental protection tasks [23,79,80,81]. While pursuing common interests, stakeholders must also share the costs of environmental protection and economic development [82,83,84]. Therefore, the establishment of such a mechanism can promote fair treatment of stakeholders, which is a crucial foundation for trust. The responsibility constraint mechanism includes three aspects: (a) ensuring the availability of funds for protecting the ecosystem, (b) implementing a penalty system for environmental damage, and (c) measuring environmental quality. The allocation and use of funds are usually means by which the management unit controls the execution department to achieve management goals. Government departments were formerly the most important management entity [22]. Through budget allocation, a government department can impose constraints on executive departments [85]. However, in the proposed environmental co-governance system, the initiator is not necessarily the government; this role can also be filled by an enterprise or community-based non-governmental organization. Thus, funds can be obtained through a variety of channels such as corporate social responsibility or other methods such as crowdfunding [86,87]. When funds can be obtained through more channels, more funds are thus available overall for use by representatives. This also means that more resources are available for solving problems related to environmental governance. For effective environmental governance, in addition to ensuring that funds are sufficient, the use of funds must also be monitored to ensure that they are being used appropriately [88,89]. Because environmental governance projects can have different initiators, establishing a set of common benchmarks for judging violations is crucial for minimizing environmental damage [90]. After a definition of environmental damage has been established, relevant policies and regulations can be developed and a penalty system can be formulated. Violators can be penalized using methods other than fines; the primary aim of the penalty system is to have a direct deterrent effect on units prone to causing environmental damage. Environmental quality is determined through comprehensive evaluation of the state of the environment, the results of which provide a direct indication of the effectiveness of environmental governance and highlight directions for improvement and suggestions for projects to be implemented [17]. Therefore, this dimension has four criteria, namely sufficient funds for environmental governance mechanisms (*C*_31_), allocation of funds (*C*_32_), a penalty scheme for causing environmental damage (*C*_33_), and satisfactory environmental quality assessment results (*C*_34_). On the basis of relevant literature, this study developed an evaluation framework for environmental co-governance, which is introduced in Figure 2.

### 2.2. Test of the Framework of Environmental Co-Governance

To ensure the effectiveness of the evaluation framework, the importance of its evaluation indicators needed to be tested. The test was divided into three phases, and a semi-structured questionnaire design was adopted. The goal of the first phase was to confirm whether the dimensions and criteria achieved the goal of environmental protection. The second phase involved defining the criteria. For these two phases, data were collected through an expert survey that employed a questionnaire with an open-ended question design as well as interviews to obtain a comprehensive understanding of experts’ opinions. In this study, 16 experts in the field of environmental governance were surveyed. Of these experts, nine were from the Pingxiang City Ecological Environment Bureau and were directly engaged in environmental governance, five were scholars, and two were executives of highly polluting companies. The aim was that these experts from industry, academia, and government would confirm the appropriateness of the names and definitions of indicators and the size of the framework from their various perspectives.

After completion of the first two phases, the third phase involved confirming whether each criterion was representative. For this phase, a closed-ended question design was adopted for an expert survey. Experts were asked to judge the importance of each criterion on a 5-point Likert scale where points 1–5 denote “very unimportant,” “not important,” “of no notable importance,” “important,” and “very important,” respectively. The experts’ scores were averaged for each criterion to determine whether the criteria were representative. Scores of 3 and 4 were set as thresholds. Criteria with scores higher than 4 were considered representative and were accepted, and those with scores less than 3 were considered unrepresentative and were rejected. A score of 3 or 4 was considered to indicate that a consensus had not yet been reached on the criterion, and that the survey needed to be conducted again. The results of the 16 expert questionnaires revealed that the two criteria with the lowest scores were correct and complete information (*C*_12_) and smooth joint decision-making by stakeholders (*C*_24_), which both had scores of 4; the other criteria attained scores higher than 4. Thus, the environmental co-governance framework was considered to have passed the test. The framework includes 3 dimensions and 11 criteria, as listed in Table 1.

### 2.3. BWM-mV Model and Process of its Application

The combined best–worst method (BWM) and modified Vlsekriterijumska Optimizacija I Kompromisno Resenje (VIKOR) method (called the BWM-mV model) is a hybrid technique that has applications such as the evaluation of service quality in the aviation industry [91], airport green performance [39], and the performance of cable company suppliers [92]. In the aforementioned studies, this model was used to solve the problem of evaluating and ranking alternatives among, for example, airlines, with respect to service quality; airports, with respect to greenness; and suppliers, with respect to price and quality. However, the focus of the modified VIKOR should be on providing suggestions on which of many avenues of improvement should be pursued. That is, the core spirit of this model is to achieve the “evaluation of alternatives and improvement suggestions more accurately [43]”. The BWM is a technique for calculating indicator weights through pairwise comparison. With this technique, decision-makers can understand the priority issues that resource-constrained cities must master in the environmental co-governance system. On this basis, the modified VIKOR method can be employed to ascertain the gap between actual performance and aspired-to performance to alert decision-makers to areas for improvement in a city’s current environmental governance system. Finally, by combining the results of the two techniques, an “important but poor performance” indicator can be found, and a path for improvement can be provided for the environmental governance of resource-constrained cities, which was the principal goal of this study.

**Figure 3:**
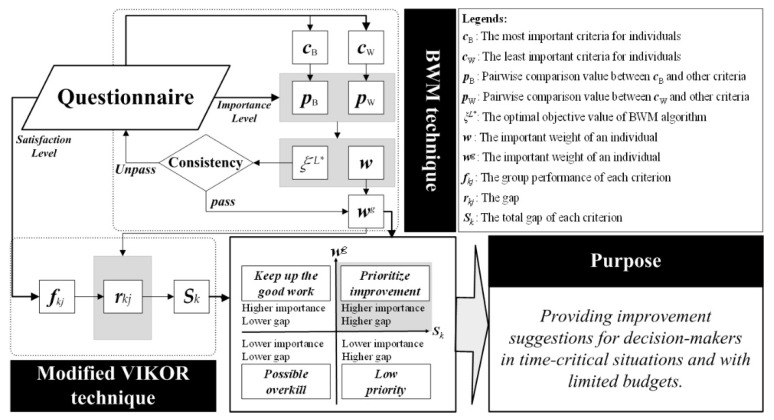
Process of applying the BWM-mV model.

This means the BWM-mV model can effectively provide suggestions for improvements to decision makers who are under time and financial constraints. The process of applying the BWM-mV model is illustrated in Figure 3.

The questionnaire was designed according to the environmental co-governance framework, which contains two types of information. The first is the importance of each criterion, which is determined by asking the interviewee to select the most important (*C_B_*) and least important criteria (*C_W_*) from all of the criteria. Next, each criterion is compared with the most important criterion (*P_B_*) and the least important criterion (*P_W_*) in pairs to obtain a ratio. A scale of 1–9 was adopted for the questionnaire used in this step. The third step was to use the BWM algorithm to identify the criterion with the highest importance weight (*w*) under the minimum ξ^L*^. Finally, ξ^L*^ is used to check the consistency. The test value is referred to as the consistency index (CI) value. If the CI value does not meet the specified threshold (0.1), then a problem exists with the raw data and the data need to be evaluated. If the CI value does meet the threshold, then the weight value of the criterion is considered reliable. In the next step, each individual weight is averaged to obtain the group weight (*w^g^*). The second type of information is the degree of satisfaction of the criteria, which reflects the current performance of the alternative criterion. The first step is to survey the respondents to determine the performance of the alternatives for each criterion. The questionnaire scale used in this step was 0–10, which is a ratio scale in which 0 points represents the most dissatisfactory and 10 points represents the most satisfactory. Subsequently, the survey results of all respondents are aggregated to obtain the average for evaluating group performance (*f_kj_*). The upper bound of the questionnaire represents the aspiration level (*f^aspired^*), and the lower bound represents the worst level (*f^worst^*). Group performance is then normalized to calculate the distance between the performance of each criterion and the aspiration level, referred to herein as *the gap* (*r_kj_*). After the gap of each criterion is obtained, the group utility (*S_k_*) is generated through integration of the weight. The steps of the BWM-mV model are detailed in Appendix A.

After the data are calculated, they are visualized in two dimensions. The horizontal axis is the gap, which represents the room for improvement of each criterion, and the vertical axis is the importance weight, which represents the importance of each criterion. The resulting graph, referred to as the *importance-gap graph*, is divided into four quadrants based on these two axes. The criteria in the first quadrant have a higher importance and a larger gap. Because of this larger gap, a performance level further from the aspiration level indicates greater room for improvement for a given criterion. Therefore, when a decision must be made with limited time and resources, the criteria in this quadrant should be improved first. The criteria in the second quadrant have a high importance and small gap (i.e., better performance). Therefore, the operation mode of these criteria should be considered to ensure that these criteria maintain adequate performance. The criteria in the third quadrant have a low importance and small gap; although these criteria have adequate performance, their importance is relatively low. Therefore, decision makers can reallocate resources by reviewing and improving resource use efficiency. The criteria in the fourth quadrant have a low importance and a large gap. Because the importance and gap of these criteria are relative conditions, these criteria are not necessarily insignificant or poorly implemented; rather, decision makers must make more effective choices under time and resource constraints. Therefore, these criteria are secondary rather than primary considerations for decision makers when improving their alternative criteria.

## 3. Empirical Analysis of Pingxiang, China

Pingxiang is a resource-constrained city in China. The environmental policies enacted by the central government require that urgent improvements be made to protect the environment. Therefore, this study applied the environmental co-governance system to help Pingxiang City Government solve this problem.

### 3.1. Resource-Constrained City: Pingxiang, Jiangxi Province, China

**Figure 4:**
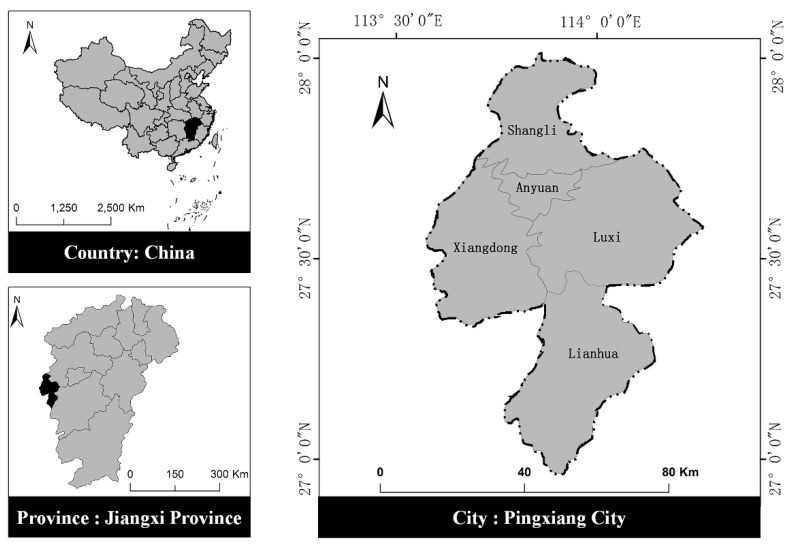
Geographical characteristics and location of Pingxiang, Jiangxi Province, China.

For a case study, we selected Pingxiang, which is a prefecture-level city located in western Jiangxi Province, China. Pingxiang has two districts (Anyuan, Xiangdong) and three counties (Luxi, Shangli, and Lianhua) (Figure 4). The establishment of Pingxiang was based on the presence of coal, but the diminishment of coal resources has caused hardship for related industries and affected local economic development. According to the Pingxiang City Statistical Yearbook 2018, Pingxiang’s fiscal deficit is growing year by year (Table 2) while its six pillar industries (i.e., mining, petrochemicals, fireworks, cement, electro-ceramics, and steel) continue to substantially contribute to pollution levels, causing enormous damage to the urban environment. Therefore, Pingxiang is a typical city under the dual pressure of: (1) having limited resources and (2) requiring improvements to reduce levels of environmental pollution; thus, multi-stakeholder collaborative governance is necessary for conserving resources and improving the urban environment. A more detailed explanation of the actual situation, based on the index system of the ECAF, is provided as follows.

#### 3.1.1. Atmosphere Conducive to the Proactive Provision of Information (*C*_11_)

Relevant policies and regulations can impose mandates on stakeholders, forcing them to disclose environmental information. Pingxiang has also increased the publicity of relevant policies to obtain more extensive environmental information. Community workers and volunteers have visited industrial parks, enterprises, and residential areas to publicize environmental protection policies, explain relevant reward and punishment measures, and encourage enterprises and residents to provide environmental information of their own volition.

#### 3.1.2. Correct and Complete Information (*C*_12_)

Pingxiang has formulated and implemented measures targeting government officials and enterprises and clearly stipulated consequences for tampering with, forging, or concealing information related to the ecological environment. To ensure the correctness of the environmental information provided by enterprises, the government has also arranged for a special investigative team to visit enterprises and confirm the correctness and completeness of the information provided through field investigation.

#### 3.1.3. High-Quality Information Communication Platform (*C*_13_)

Pingxiang currently has numerous environmental information communication platforms. These include a government website, social networking platform, telephone hotline, and petition mailbox. The primary function of the government website and social networking platform is to publish environmental information in a timely manner. These platforms also provide a basis for “online surveys”, “environmental consultation”, and “solicitation of opinions” projects. Citizens can also make use of these functions to ask questions and express their opinions on environmental issues. The hotline and petition mailbox were established to enable citizens to file complaints regarding environmental problems. When a complaint is received, the Pingxiang City Ecological Environment Bureau (PCEEB) follows up and addresses the concern in a timely manner, and the results of implementing measures to resolve the relevant problem are provided to the individual who filed the original complaint.

#### 3.1.4. Diverse Environmental Governance Mechanisms (*C*_21_)

The government is the main sponsor of environmental actions, such as the introduction of policies to guide the implementation of environmental protection measures. In addition, enterprises or industry associations often initiate various environmental protection measures. For example, the Jiangxi Porcelain Chamber of Commerce has brought together all of Pingxiang’s electroceramics enterprises for joint research and development, which has not only promoted the upgrading of common electroceramics products to high-tension electroceramics products but has also effectively reduced the amount of environmental pollution emitted during the production process. Citizens rely heavily on volunteer groups to launch environmental initiatives. Pingxiang has more than 200,000 registered volunteers and conducts urban waste clean-up operations of increasingly large scale every week.

#### 3.1.5. Effective Environmental Protection Projects (*C*_22_)

Being able to confirm whether environmental protection projects can truly achieve the goal of protecting or improving the condition of the ecological environment is crucial. Therefore, a perfect project management system is vital for managing various stages of projects to achieve comprehensive management before, during, and after implementation. Evaluation of the effectiveness of projects in Pingxiang is mainly performed by an entrusted third-party consulting firm. According to relevant regulations, a project with a total investment of more than CN¥30 million must be put out for public bidding, and a project with a total investment of less than CN¥30 million but with a single environmental project of more than CN¥500,000 must also be put out for public bidding. Only one local company currently holds an environmental project evaluation qualification. Therefore, the evaluation of most projects must be performed by consulting companies outside of Pingxiang.

#### 3.1.6. Robust Co-Management and Monitoring Mechanisms (*C*_23_)

A part-time supervisory team has been established in Pingxiang. The panel is composed of government workers, business managers, ordinary citizens, academics, and journalists. The team drafts an annual supervision plan, supervises implementation through field investigation, conducts interviews to relay problems identified during oversight actions, and proposes improvements. The results of improvement measures are also reexamined to ensure that problems are resolved.

#### 3.1.7. Smooth Joint Decision-Making by Stakeholders (*C*_24_)

Pingxiang employs a political consultative system. Members of the National Committee of the Chinese People’s Political Consultative Conference (CPPCC) come from all walks of life and are selected through consultation and recommendation. Members of the CPPCC must first have a broad understanding of the aspirations and demands of the public and then make suggestions and recommendations to the government in the form of research reports, proposals, and recommendations.

#### 3.1.8. Sufficient Funds for Environmental Governance Mechanisms (*C*_31_)

Taxes and higher-level government grants are Pingxiang’s main sources of funding to tackle environmental problems. In 2017, Pingxiang’s tax revenue was CN¥6684.03 million, of which CN¥1238.83 million (18.53%) was from direct expenditure on environmental protection. To raise more funds, environmental projects have taken flexible forms. For example, the “Sponge City” project is based on the public–private partnership model. This model broadens the available financing channels and attracts substantial private capital for project investment. Additionally, the use of a variety of financial instruments (e.g., green credit, green bonds, and green insurance) has further enriched environmental financing.

#### 3.1.9. Allocated Funds (*C*_32_)

The use of funds for environmental projects is publicly disclosed on the government’s environmental information platform. The audit department audits the use of funds on a regular basis and deals with situations involving the illegal use of funds. The government supervisory team also conducts investigations to monitor the use of environmental funds. For example, in 2019, the PCEEB spent CN¥24 million on energy conservation and environmental protection projects, and the implementation rate was approximately 98% of the budget available at the beginning of the year.

#### 3.1.10. Penalty for Causing Environmental Damage (*C*_33_)

Pingxiang City Government has implemented various punishment measures (e.g., production restrictions, production suspension and rectification, heavy fines, and closure) to penalize enterprises for discharging wastewater, waste gas, and solid waste and emitting noise pollution. In 2017, Pingxiang had 200 environmental violations on file, accounting for 9.9% of the total number of cases in Jiangxi Province. In the same year, the city government issued cumulative fines of CN¥52.54 million, but the actual amount received was CN¥9.11 million. In addition to these punishment measures, the government website also discloses the environmentally damaging behavior of enterprises. Pingxiang has also enacted regulations regarding the city’s appearance and environmental hygiene, and citizens who violate the regulations face penalties of up to CN¥2000.

#### 3.1.11. Satisfactory Environmental Quality Assessment Results (*C*_34_)

Numerous environmental monitoring projects are currently underway in Pingxiang, including 51 ambient air and waste gas monitoring projects; 86 surface water and wastewater monitoring projects; 22 soil, water sediment, and solid waste monitoring projects; 4 biological monitoring projects; and 6 noise and vibration monitoring projects. The environmental quality of Pingxiang has recently improved considerably through the strengthening of environmental governance and expansion of the scope of monitoring. For the four consecutive months from May to August 2020, Pingxiang was ranked first among the cities in Jiangxi Province in terms of air quality. The water quality level of most bodies of water has been maintained at “good” or above.

### 3.2. Data Collection and Analysis of the Results

Data collection was divided into two parts. For the first part, which was based on the operating process of the BWM, 10 experts were invited to determine the best and worst factors on the basis of their experience and expertise, as shown in Table 3, Table 4 and Table 5 (In this process, the dimension part served as an example. The questionnaire design and collection and analysis of the data are detailed in the Appendix B and Appendix C). The survey participants were managers of government environmental departments and scholars in the field of environmental research.

The questionnaire for the technical stage, which involved the application of the VIKOR method, was completed by relevant stakeholders, including 15 environmental department managers (EDMs), 23 business executives (BEs), and 50 citizens (see Table 6). The data source for the performance value of each criterion was the judgment of the stakeholders. In some cases, only certain stakeholders could ascertain whether certain criteria were satisfied; thus, some items were only answered by these stakeholders. For example, citizens cannot evaluate criteria *C*_31_, *C*_32_, and *C*_33_; BEs cannot evaluate criteria *C*_31_ and *C*_34_; and EDMs cannot evaluate criteria *C*_10_ and *C*_11_. In terms of average performance, the best performance criterion was sufficient funds for environmental governance mechanisms (*C*_31_), followed by correct and complete information (*C*_12_) and allocated funds (*C*_32_).

The calculation results of the BWM-mV model are presented in Table 7. Of the dimensions, effectiveness of and engagement in environmental co-governance actions (*D*_2_) was the most important (0.504). The second most important was correctness and fluidity of public information (*D*_1_), with a weight of 0.313, followed by the effect and binding force of environmental governance mechanisms (*D*_3_), with a weight of 0.184. The criteria were ranked as follows in descending order of importance: *C*_21_, *C*_24_, *C*_23_, *C*_13_, *C*_11_, *C*_12_, *C*_32_, *C*_33_, *C*_22_, *C*_34_, and *C*_31_. The dimension with the largest gap was the effect and binding force of environmental governance mechanisms (*D*_3_), followed by effectiveness of and engagement in environmental co-governance actions (*D*_2_) and correctness and fluidity of public information (*D*_1_). The criteria were ranked as follows in descending order of gap value: *C*_33_, *C*_22_, *C*_24_, *C*_13_, *C*_34_, *C*_23_, *C*_11_, *C*_21_, *C*_12_, *C*_32_, and *C*_31_.

A Cartesian coordinate system was obtained using the gap and global weight values as the X-axis and Y-axis, where the intersection represents the average value. The results (see Table 8) are presented in four quadrants. High-quality information communication platform (*C*_13_) and smooth joint decision-making by stakeholders (*C*_24_) belong to the first quadrant. Atmosphere conducive to the proactive provision of information (*C*_11_), diversified environmental governance mechanisms (*C*_21_), and robust co-management and monitoring mechanisms (*C*_23_) belong to the second quadrant. Correct and complete information (*C*_12_), sufficient funds for environmental governance mechanisms (*C*_31_), and allocated funds (*C*_32_) belong to the third quadrant. Effective environmental protection projects (*C*_22_), penalty for causing environmental damage (*C*_33_), and satisfactory environmental quality assessment results (*C*_34_) belong to the fourth quadrant.

### 3.3. Discussion

**Figure 5:**
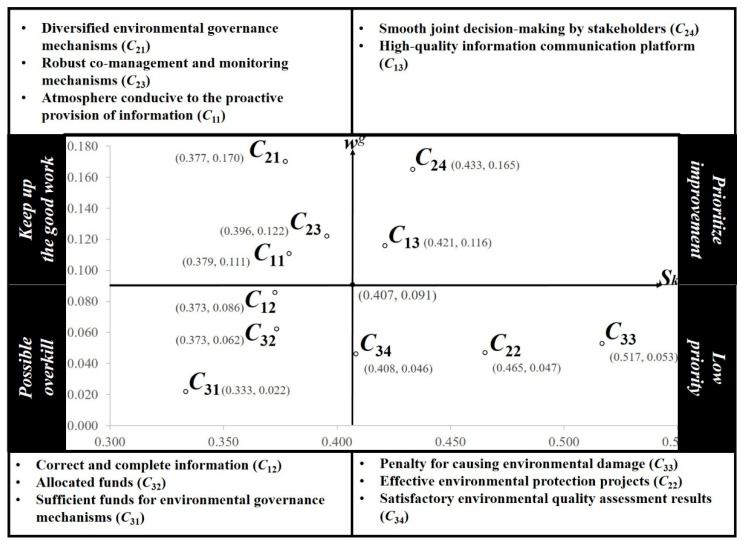
Importance-gap graph of the criteria.

In the importance-gap graph of the criteria, the criteria are distributed across different quadrants, and each quadrant has different development strategies (see Figure 5). The following discussion of improvement strategies is based on the criteria for each quadrant.

#### 3.3.1. First Quadrant

The first quadrant contains two criteria, namely high-quality information communication platform (*C*_13_) and smooth joint decision-making by stakeholders (*C*_24_).

Regarding the criterion of high-quality information communication platform (*C*_13_), Pingxiang lacks a professional platform for smooth and effective communication between stakeholders, despite it being a crucial criterion. Pingxiang’s existing platforms have some problems, such as a lack of information integration, low levels of information exchange, and low levels of specialization. Therefore, Pingxiang City Government should develop a professional communication platform for environmental information as soon as possible. This platform should integrate all environmental information and have multiple functions, such as information disclosure, discussion of environmental matters, advice sharing, and filing of complaints. When the platform has become operational, the government should vigorously publicize and promote it to improve the utilization rate of the platform and thereby enable stakeholders to conveniently exchange information.

Smooth joint decision-making by stakeholders (*C*_24_) is also hindered by the lack of an adequate information communication platform. Although CPPCC members are responsible for collecting citizens’ opinions and suggestions, the amount they collect is extremely limited. Therefore, the development of a communication platform for environmental information is highly important. A strong communication platform would enable more people to participate in environmental decision-making and better enable them to express their opinions and offer suggestions.

Therefore, Pingxiang’s first priority in terms of environmental governance should be to integrate its decentralized systems and platforms as quickly as possible, building a trust-based professional platform for stakeholders to share information, communicate, make decisions, and supervise each other to achieve environmental protection. Being able to mobilize existing resources is paramount for improving the effectiveness of the platform. The platform should therefore be at a higher level in the original administrative organization structure, which would also increase the willingness of stakeholders to trust in and engage with the platform. Building trust helps in reaching a consensus and can facilitate joint decision-making. Public disclosure and transparency of information are crucial for forming a foundation for trust because it is critical that all stakeholders believe that rules are in place for addressing problems. Pingxiang City Government should develop a transparent and public platform for environmental governance, and this platform must be at a high administrative level and ultimately ensure that all participating stakeholders adhere to certain rules.

#### 3.3.2. Second Quadrant

The second quadrant contains three criteria, namely atmosphere conducive to the proactive provision of information (*C*_11_), diversified environmental governance mechanisms (*C*_21_), and robust co-management and monitoring mechanisms (*C*_23_).

Regarding the criterion of an atmosphere conducive to the proactive provision of information (*C*_11_), under the current information disclosure mechanism in Pingxiang, enterprises must disclose their own environmental information under the policies in place. Additionally, this mechanism incudes a reward policy that encourages citizens to actively provide environmental pollution information. Although this mechanism has achieved some positive results, it still requires improvement. Enterprises occasionally still conceal environmental information, and only a small group of citizens care about the environment. Therefore, the government should focus on the establishment and improvement of the care mechanism. The government can foster the interest of enterprises and citizens in environmental matters by providing more environmental consultation, organizing more popular science activities concerning environmental knowledge, and helping enterprises to implement practical solutions to environmental problems. Such measures can enhance the environmental awareness of enterprises and citizens, thereby alerting them to the importance of environmental information and increasing their willingness to offer information.

Regarding the criterion of diversified environmental governance mechanisms (*C*_21_), Pingxiang City Government is the main sponsor of environmental initiatives, and relevant actions are occasionally organized by enterprises and citizens. However, a truly diverse set of environmental governance mechanisms has not yet been established. Enterprises and citizens may be more concerned with the legitimacy of their actions; consequently, the environmental initiatives they organize are not only few in number but also weak in impact. Therefore, Pingxiang should develop a policy for initiating environmental action. This policy must be able to provide guidance for enterprises and citizens to initiate environmental action while safeguarding the legitimacy of their actions.

Regarding the criterion of robust co-management and monitoring mechanisms (*C*_23_), the measures taken by Pingxiang’s part-time supervisory team have been highly effective and well covered by the media. However, addressing all environmental problems is impossible due to the limited number of panel members. Therefore, more stakeholders should be involved in mutual decision-making and mutual supervision. Informatization is one approach that can help to meet this goal. Establishing an adequate professional platform for sharing environmental information can not only help in meeting the criteria in the first quadrant but also increase the convenience of participating in environmental supervision.

Because the criteria in the second quadrant have greater importance than those in other quadrants, Pingxiang should ensure the proper functioning of the information disclosure, action, and supervisory mechanisms. However, the criteria in the second quadrant also indicate that the gap is relatively small. Therefore, the key to ensuring the proper functioning of these mechanisms is to adjust the relevant internal resources and development objectives of each criterion without reducing the input of existing resources. The ultimate goal of these adjustments is to improve the efficiency of resource utilization. Therefore, more resources should be used for establishing and improving a “care mechanism” to promote interest in the environment, an environmental action initiation mechanism, and an information-based supervision platform. A well-developed care mechanism would make it easier for stakeholders to form partnerships based on mutual trust. This would significantly reduce the amount of environmental information withheld, resulting in a better atmosphere for the active provision of information. Such a care mechanism can also promote the initiation of environmental actions by enterprises and citizens. Moreover, because the action mechanism offers an institutional guarantee, enterprises and citizens should be willing to initiate such environmental actions and should also have the opportunity to obtain corresponding benefits. This can change the status quo, which currently depends mainly on the government’s initiation of environmental actions, and gradually form a variety of environmental governance mechanisms. This care mechanism can also prompt enterprises and citizens to participate in environmental supervision. Moreover, the establishment of an information-based supervision platform would not only encourage stakeholders to be more attentive but would also make it more convenient for them to participate in environmental supervision.

#### 3.3.3. Third Quadrant

The third quadrant contains three criteria, namely correct and complete information (*C*_12_), sufficient funds for environmental governance mechanisms (*C*_31_), and allocated funds (*C*_32_).

Regarding the criterion of correct and complete information (*C*_12_), Pingxiang City Government has implemented strict measures to punish those responsible for falsifying environmental information and has conducted environmental information investigations. These measures make the gap for this criterion relatively small, but do not guarantee that all information is correct and complete. To improve the accuracy and integrity of information, Pingxiang requires a more convenient information exchange platform and must enforce punishments more strictly.

Regarding the criterion of sufficient funds for environmental governance mechanisms (*C*_31_), despite having limited financial resources, Pingxiang City Government still increased its investment in environmental governance, meaning that the gap for this criterion is relatively small. This result actually depends on China’s assessment system for local officials. China has included environmental indicators in its assessment of officials, resulting in a substantial increase in environmental funding in all cities. Compared with other cities, Pingxiang has a relatively small total amount of environmental investment. Therefore, in the future, Pingxiang should raise funds more broadly for environmental protection.

Regarding the criterion of allocated funds (*C*_32_), strict investigations and audit measures have increased Pingxiang’s implementation rate for environmental funds, meaning that this criterion also has a relatively small gap. In the future, in addition to paying attention to the implementation rate of funds, Pingxiang should pay more attention to the effects of using funds. Therefore, the government should require the construction unit of environmental projects to set clear objectives for each construction phase and evaluate the achievement of these objectives.

Because of the positive impact of China’s assessment system for local officials, the number of cases of falsification of environmental information in Pingxiang has decreased significantly in the past period, and the investment in environmental governance has increased year by year. However, the total amount of environmental funding remains low, and the effectiveness of its use remains unclear. Therefore, Pingxiang City Government should request that the construction unit of each project formulate detailed and clear objectives for each construction phase and determine the effectiveness of the use of funds on the basis of the actual achievement of the objectives. In the long run, Pingxiang must also open up a wider range of funding channels to raise more money for environmental protection. In general, the criteria in the third quadrant have relatively small gaps and are of low importance. Therefore, the aforementioned improvements are not urgently required and should be deferred to the next phase, in which they can be foci.

#### 3.3.4. Fourth Quadrant

The fourth quadrant contains three criteria, namely effective environmental protection projects (*C*_22_), penalty for causing environmental damage (*C*_33_), and satisfactory environmental quality assessment results (*C*_34_).

Regarding the criterion of effective environmental protection projects (*C*_22_), environmental projects in Pingxiang are mainly assessed by environmental impact assessment (EIA) companies. An EIA company is entrusted by the construction unit of a given project to examine the feasibility and effectiveness of the project and issue an EIA report. However, there are a large number of EIA companies in China, and the primary goal of these companies is to make a profit. The respective interests of EIA companies and project construction units make it easy for the two parties to “cooperate” with each other. In fact, EIA companies must issue a “project feasibility” report to obtain a commission from the project construction unit—this has become one of the industry’s unspoken rules. The reality in Pingxiang is that these companies judge all environmental projects to have “project feasibility,” which raises doubts regarding whether these projects will actually achieve their environmental goals. This is also the direct cause of the low level of satisfaction for this criterion. Therefore, Pingxiang should ensure the authenticity and accuracy of EIA reports. The government should perform strict examinations of the reports issued by EIA companies and, if necessary, arrange for a review team to conduct a thorough examination of the project. Additionally, according to the results of the examinations, the government should severely penalize EIA companies that produce false reports.

The main problem in Pingxiang regarding the criterion of penalties for causing environmental damage (*C*_33_) is the lack of enforcement of punishments for environmental violations. Pingxiang has strict environmental regulations and high fines for harming the environment, but the actual rate of fines paid is extremely low. This indicates that law enforcement is not strong enough to truly deter environmental damage. Pingxiang should therefore improve law enforcement. As a concrete measure, the government should expose enterprises and individuals who delay paying or refuse to pay fines and add the executives of the related enterprises to the credit blacklists to achieve a real deterrent effect.

Regarding the criterion of satisfactory environmental quality assessment results (*C*_34_), Pingxiang’s ranking among the cities of Jiangxi Province in terms of environmental quality has recently improved year by year. However, its ranking nationwide indicates that it is still a long way from being regarded as a city with good environmental quality. In terms of air quality, which is a primary concern for citizens, Pingxiang’s PM_2.5_ index has long remained stable at approximately 80–100, which is still a long way from being considered “excellent” (0–50). Therefore, Pingxiang should not be satisfied with its ranking within the province but should rather take the best environmental quality as a benchmark for improvements.

Therefore, Pingxiang should also ensure the authenticity and accuracy of EIA reports. Strict examinations of EIA reports and investigation and prosecution of EIA companies that issue false reports would reduce the falsification behavior of EIA companies and further improve the authenticity and accuracy of reports. This would help to truly distinguish between “good” and “bad” environmental projects, and the evaluation results can be used to implement incentives and penalties. Specific punishments include issuing a large fine or blacklisting the individual managing the enterprise. Moreover, the government should enhance law enforcement to improve the effects of punishments. In general, the criteria in the fourth quadrant are of low importance. The aforementioned improvements are therefore not urgently required and should be deferred to the next phase, in which they can be foci.

In summary, with the concerted efforts of all stakeholders, environmental governance in Pingxiang has achieved some success, but it still requires improvement. The key to successful environmental governance in Pingxiang, as a resource-constrained city, lies in the rational allocation and efficient use of resources. After a comprehensive analysis of the importance and size of the gap of each criterion, this study concluded that improvements should be divided into four stages. The first stage is to establish a public and transparent environmental platform. The second stage is to maintain the effective operation of mechanisms for environmental information disclosure, environmental actions, and supervision. The third stage is to improve the supervision of environmental projects and enhance the enforcement of penalties for causing environmental damage. The fourth stage is to improve the efficiency of the use of environmental funds. Organizing the four phases would contribute to a new realignment of resources and more efficient use of resources on the basis of trust. The allocation of more resources to the establishment of an environmental platform, which would give the city a higher ranking in environmental quality and enable stakeholders to share information in an environment based on mutual trust, is urgently required for Pingxiang. After the platform has been established, the environment of trust it offers can facilitate the development of a caring mechanism. This should lead to the formation of partnerships between various stakeholders, who are more willing to participate in and proactively initiate environmental actions and conscious environmental supervision. This will enable more efficient use of resources as well as help in the third phase to improve the provision of surplus resources. Further improvements in environmental governance will lead to improved effectiveness of the stakeholder co-governance mechanism and better use of resources. More resources can then be devoted to the fourth stage of improvement. The fourth phase aims to improve the efficiency regarding how funds are spent; such improvement can further enhance resource usage efficiency, thus realizing a virtuous cycle and enabling Pingxiang to effectively achieve its environmental protection goals.

## 4. Conclusions

Protecting and improving the environment require not only a large amount of capital investment but also the cooperation of stakeholders. Therefore, the purpose of this study was to provide effective improvement suggestions for cities with limited resources from the perspective of environmental co-governance. Researchers conducting follow-up studies may discuss local environmental protection issues based on the operation mode of this research.

This study makes three main contributions. The first is the development of an assessment framework for environmental co-governance, consisting of 3 dimensions and 11 criteria. The second is the establishment of a mechanism for analyzing and visualizing environmental co-governance. The third is the analysis of Pingxiang City and proposal of areas for improvement. The results indicate that diverse environmental governance mechanisms (*C*_21_) constitute the most critical criterion for effective environmental co-governance, followed by smooth joint decision-making by stakeholders (*C*_24_) and robust co-management and monitoring mechanisms (*C*_23_). The criteria are ranked as follows in descending order of importance: *C*_21_, *C*_24_, *C*_23_, *C*_13_, *C*_11_, *C*_12_, *C*_32_, *C*_33_, *C*_22_, *C*_34_, and *C*_31_. The first quadrant contains two criteria, namely high-quality information communication platform (*C*_13_) and smooth joint decision-making by stakeholders (*C*_24_). The second quadrant contains three criteria, namely an atmosphere conducive to the proactive provision of information (*C*_11_), diverse environmental governance mechanisms (*C*_21_), and robust co-management and monitoring mechanisms (*C*_23_). The third quadrant contains three criteria, namely correct and complete information (*C*_12_), sufficient funds for environmental governance mechanisms (*C*_31_), and allocated funds (*C*_32_). The fourth quadrant contains three criteria, namely effective environmental protection projects (*C*_22_), penalty for causing environmental damage (*C*_33_), and satisfactory environmental quality assessment results (*C*_34_). In conclusion, the principal finding is that a high-quality information communication platform (*C*_13_) and smooth joint decision-making by stakeholders (*C*_24_) require the most urgent improvement by the Pingxiang City Government. A transparent and public platform must be developed for environmental governance, and this platform must operate at a high administrative level and ultimately ensure that all stakeholders adhere to certain rules.

Although the environmental co-governance system has many functions, this research has two limitations. The first is the scope of planning. Co-governance is one type of governance model based on a network structure. In cases with a broad scope of planning, co-governance may be combined with other governance models (e.g., top-down or bottom-up modes). The second limitation is the administrative regions considered. Because the focus of this indicator framework is on co-governance, collaboration across administrative regions and local administrative organization class was not considered during the development of the framework. Therefore, future research may expand this indicator framework to include such collaboration.

## Figures and Tables

**Table 1:** Environmental co-governance framework.

Dimensions	Criteria	Descriptions
Correctness and fluidity of public information (*D*_1_)	Atmosphere conducive to the proactive provision of information (*C*_11_)	Atmosphere refers to the feeling generated by each stakeholder through the operation of a mechanism that must be able to encourage stakeholders to actively and willingly share their information.
	Correct and complete information (*C*_12_)	This criterion means that the information provided by stakeholders must be correct and complete in addition to being useful for environmental protection.
	High-quality information communication platform (*C*_13_)	This criterion refers to a platform that enables stakeholders to exchange information in a timely and convenient manner.
Effectiveness of and engagement in environmental co-governance actions (*D*_2_)	Diversified environmental governance mechanisms (*C*_21_)	This criterion refers to related actions (e.g., policies, regulations, mechanisms, or projects) proposed by different sponsors for environmental protection or improvement.
	Effective environmental protection projects (*C*_22_)	This criterion reflects whether the implemented project can achieve the purpose of protecting or improving the ecological environment.
	Robust co-management and monitoring mechanisms (*C*_23_)	This criterion refers to whether the management model, regulations, and related supervision mechanisms have clear specifications.
	Smooth joint decision-making by stakeholders (*C*_24_)	This criterion represents adequate communication and coordination between stakeholders, through which a mutual decision can be reached.
The effect and binding force of environmental governance mechanisms (*D*_3_)	Sufficient funds for environmental governance mechanisms (*C*_31_)	This criterion refers to whether the available funds are sufficient for solving environmental governance problems.
	Allocated funds (*C*_32_)	This criterion refers to the use of funds at every stage of the project; confirming whether the expected outcome of using allocated funds has been met is crucial.
	Penalty for causing environmental damage (*C*_3__3_)	A penalty system should be introduced with the goal of preventing environmental damage. In addition to fines, other penalties should be employed to act as additional deterrents. Finally, the penalties must be clearly stipulated in relevant regulations.
	Satisfactory environmental quality assessment results (*C*_34_)	This criterion refers to whether the results of checking and verifying the current state of the environment are satisfactory from a comprehensive perspective.

**Table 2:** Government revenue and expenditure of Pingxiang, 2007–2017.

Year	Government Revenue (Million Yuan)	Government Expenditure (Million Yuan)	Balance (Million Yuan)
2007	3307.73	3514.64	−206.91
2008	4133.27	5085.46	−952.19
2009	4693.53	6963.53	−2270.00
2010	6407.95	8669.77	−2261.82
2011	8692.43	10,871.43	−2179.00
2012	10,050.52	13,434.49	−3383.97
2013	10,979.73	14,960.07	−3980.34
2014	11,705.53	15,654.83	−3949.30
2015	13,048.32	18,492.70	−5444.38
2016	13,563.83	20,001.97	−6438.14
2017	14,616.21	22,493.77	−7877.56

The data were collected from the Pingxiang City Statistical Yearbook.

**Table 3:** Most and least important factors selected.

Expert No.	1	2	3	4	5	6	7	8	9	10
Most important	*D*_2_	*D*_2_	*D*_2_	*D*_2_	*D*_2_	*D*_2_	*D*_2_	*D*_2_	*D*_2_	*D*_2_
Least important	*D*_3_	*D*_3_	*D*_1_	*D*_3_	*D*_3_	*D*_3_	*D*_1_	*D*_3_	*D*_3_	*D*_3_

**Table 4:** Obtained best-to-others vector.

Expert No. 1	*D*_1_	*D*_3_	Expert No. 6	*D*_1_	*D*_3_
*D*_2_	2	4	*D*_2_	2	3
Expert No. 2	*D*_1_	*D*_3_	Expert No. 7	*D*_1_	*D*_3_
*D*_2_	1	3	*D*_2_	3	2
Expert No. 3	*D*_1_	*D*_3_	Expert No. 8	*D*_1_	*D*_3_
*D*_2_	3	3	*D*_2_	1	2
Expert No. 4	*D*_1_	*D*_3_	Expert No. 9	*D*_1_	*D*_3_
*D*_2_	1	4	*D*_2_	2	4
Expert No. 5	*D*_1_	*D*_3_	Expert No. 10	*D*_1_	*D*_3_
*D*_2_	2	4	*D*_2_	1	1

**Table 5:** Obtained others-to-worst vector.

Expert No. 1	*D*_3_	Expert No. 2	*D*_3_	Expert No. 3	*D*_1_	Expert No. 4	*D*_3_	Expert No. 5	*D*_3_
*D*_2_	4	*D*_2_	3	*D*_2_	3	*D*_2_	4	*D*_2_	4
*D*_1_	3	*D*_1_	2	*D*_3_	2	*D*_1_	3	*D*_1_	3
Expert No. 6	*D*_3_	Expert No. 7	*D*_1_	Expert No. 8	*D*_3_	Expert No. 9	*D*_3_	Expert No. 10	*D*_3_
*D*_2_	3	*D*_2_	3	*D*_2_	2	*D*_2_	4	*D*_2_	1
*D*_1_	2	*D*_3_	2	*D*_1_	2	*D*_1_	3	*D*_1_	1

**Table 6:** Performance of stakeholders.

Criteria	EDM	BE	Citizen	Average
Atmosphere conducive to the proactive provision of information (*C*_11_)	7.500	5.944	5.917	6.212
Correct and complete information (*C*_12_)	7.583	5.944	6.000	6.273
High-quality information communication platform (*C*_13_)	7.750	6.000	5.028	5.788
Diversified environmental governance mechanisms (*C*_21_)	7.917	5.722	5.917	6.227
Effective environmental protection projects (*C*_22_)	7.667	6.611	3.944	5.348
Robust co-management and monitoring mechanisms (*C*_23_)	8.000	6.833	5.000	6.045
Smooth joint decision-making by stakeholders (*C*_24_)	7.583	5.778	4.972	5.667
Sufficient funds for environmental governance mechanisms (*C*_31_)	6.667	-	-	6.667
Allocated funds (*C*_32_)	6.833	5.889	-	6.267
Penalty for causing environmental damage (*C*_33_)	-	4.833	-	4.833
Satisfactory environmental quality assessment results (*C*_34_)	-	-	5.917	5.917

EDM = Environmental Department Manager; BE = Business Executive.

**Table 7:** Calculation results of the BWM-mV model.

Dimensions/Criteria	Local Weights	Global Weights	Performance	Gap
**Correctness and fluidity of public information (*D*_1_)**	**0.313**		**6.072**	**0.393**
Atmosphere conducive to the proactive provision of information (*C*_11_)	0.355	0.111	6.212	0.379
Correct and complete information (*C*_12_)	0.275	0.086	6.273	0.373
High-quality information communication platform (*C*_13_)	0.371	0.116	5.788	0.421
**Effectiveness of and engagement in environmental co-governance actions (*D*_2_)**	**0.504**		**5.918**	**0.408**
Diversified environmental governance mechanisms (*C*_21_)	0.338	0.170	6.227	0.377
Effective environmental protection projects (*C*_22_)	0.093	0.047	5.348	0.465
Robust co-management and monitoring mechanisms (*C*_23_)	0.242	0.122	6.045	0.396
Smooth joint decision-making by stakeholders (*C*_24_)	0.328	0.165	5.667	0.433
**The effect and binding force of environmental governance mechanisms (*D*_3_)**	**0.183**		**5.813**	**0.419**
Sufficient funds for environmental governance mechanisms (*C*_31_)	0.119	0.022	6.667	0.333
Allocated funds (*C*_32_)	0.339	0.062	6.267	0.373
Penalty for causing environmental damage (*C*_33_)	0.288	0.053	4.833	0.517
Satisfactory environmental quality assessment results (*C*_34_)	0.253	0.046	5.917	0.408
**Total performance**			**5.947**	
**Total gap**				**0.405**

**Table 8:** Importance-gap analysis of the criteria.

Dimensions/Criteria	Gap	Global Weights	Position	Quadrant
Atmosphere conducive to the proactive provision of information (*C*_11_)	0.379	0.111	**(−, +)**	**Ⅱ**
Correct and complete information (*C*_12_)	0.373	0.086	**(−, −)**	**Ⅲ**
High-quality information Communication platform (*C*_13_)	0.421	0.116	**(+, +)**	**Ⅰ**
Diversified environmental governance mechanisms (*C*_21_)	0.377	0.170	**(−, +)**	**Ⅱ**
Effective environmental protection projects (*C*_22_)	0.465	0.047	**(+, −)**	**Ⅳ**
Robust co-management and monitoring mechanisms (*C*_23_)	0.396	0.122	**(−, +)**	**Ⅱ**
Smooth joint decision-making by stakeholders (*C*_24_)	0.433	0.165	**(+, +)**	**Ⅰ**
Sufficient funds for environmental governance mechanisms (*C*_31_)	0.333	0.022	**(−, −)**	**Ⅲ**
Allocated funds (*C*_32_)	0.373	0.062	**(−, −)**	**Ⅲ**
Penalty for causing environmental damage (*C*_33_)	0.517	0.053	**(+, −)**	**Ⅳ**
Satisfactory environmental quality assessment results (*C*_34_)	0.408	0.046	**(+, −)**	**Ⅳ**
**Average of criteria gap**	**0.407**			
**Average of criteria global weights**		**0.091**		

## Data Availability

The data presented in this study are available in insert article and Appendix C here.

## Appendix A. Operating Steps of Best Worst Method–Modified VIKOR Model

This model comprises two methods, namely the best worst method (BWM) and modified VIKOR (mV) method. The BWM is a relatively new multi-criteria decision-making method [32,33,34]. The calculation steps of the BWM are presented in Steps 1–5 [93], and those of the mV model are presented in Steps 6 and 7 [43,44].

Step 1: A set of *n* assessment criteria are identified.

Step 2: According to personal preference, the assessor (expert) selects the most important ($C_{B}$) and least important ($C_{W}$) criteria among the set identified in Step 1.

Step 3: The assessor (expert) conducts pairwise comparisons between the best criterion and other criteria ($P_{B}$) by determining preferences on a scale of 1 (“equally important”) to 9 (“extremely important”). The resulting vector is as follows:

(A1) $$P_{B}=(p_{B1},p_{B2},\ldots ,p_{Bn})$$

Step 4: The assessor (expert) conducts pairwise comparisons between the worst criterion and the other criteria ($P_{W}$) by determining preferences on a scale of 1 (equally important) to 9 (extremely important). The resulting vector is as follows:

(A2) $$P_{W}=(p_{1w},p_{2w},\ldots ,p_{nw})$$

Step 5: To determine the optimal weights of the criteria $w=(w_{1}^{*},w_{2}^{*},\ldots ,w_{n}^{*})$ and $\xi^{L*}$, the maximum absolute differences $\left\{\left|w_{B}-p_{Bj}w_{j}\right|\mathrm{and}\left|w_{j}-p_{jw}w_{w}\right|\right\}$ are minimized as follows:

(A3) $$\begin{array}{l} \min\underset{j}{\max}\left\{\left|w_{B}-p_{Bj}w_{j}\right|\mathrm{and}\left|w_{j}-p_{jw}w_{w}\right|\right\}\\ s.t.\\ \sum_{j=1}w_{j}=1,\\ w_{j}\geq 0,\mathrm{for}\mathrm{all}j. \end{array}$$

This problem is transferred to the following linear programming problem:

(A4) $$\begin{array}{l} min\xi^{L}\\ s.t.\\ \left|w_{B}-p_{Bj}w_{j}\right|\leq \xi^{L}\\ \left|w_{j}-p_{jw}w_{w}\right|\leq \xi^{L}\\ \sum_{j=1}w_{j}=1,\\ w_{j}\geq 0,\mathrm{for}\mathrm{all}j. \end{array}$$

Solving this problem produces the optimal weights w and $\xi^{L*}$. $\xi^{L*}\in \left[0,1\right]$ is considered an indicator of consistency of the pairwise comparisons (i.e., values close to 0 are highly consistent). The test value is the consistency index (CI) value. If the CI value does not meet the specified threshold (0.1), then a problem exists in the raw data, which must be evaluated. If the CI value meets the threshold, then the weight value of the criterion is considered reliable.

Step 6: Steps 1–5 are repeated to obtain the local weight of each dimension and criterion. Next, the local weight of each dimension is used to obtain the global weight of each criterion, namely individual weight. Then, the individual weights are averaged to obtain the group weight (*w^g^*).

Step 7: The aspiration level (the best value) is defined as $f_{}^{aspired}$ for the *n*th criterion and the worst value is defined as $f_{}^{worst}$ for all criteria.

For the linguistic/semantic questionnaire in this study, performance scores ranging from 0 to 10 (very bad ← 0, 1, 2, …, 9, 10 → very good) are used in natural language; therefore, the highest score for the aspiration level is 10, and the score for the worst value is 0. Hence, $f_{}^{aspired}=10$ is defined as the aspiration level, and $f_{}^{worst}=0$ is defined as the worst value.

Step 8: The mean group utility for the gap is defined, after which the priority improvement strategy is established. These values can be calculated using Equation (A5):

(A5) $$s_{k}=\sum_{j=1}^{n}w_{j}r_{kj}=\sum_{j=1}^{n}w_{j}(|f_{}^{aspired}-f_{kj}|)/(|f_{}^{aspired}-f_{}^{worst}|),$$

where $s_{k}$ denotes the normalized ratio (%) of distance to the aspiration level, which implies the synthesized gap of the criteria. Here, $w_{j}$ indicates the importance of the criteria obtained from the BWM.

## Appendix B. Questionnaire for Best Worst Method–Modified VIKOR Model

The questionnaire for this model also contains two parts, namely best worst method (BWM) and modified VIKOR (mV) components.

### Appendix B.1. BWM Questionnaire

The purpose of this study is to explore the relationships among the main factors influencing environmental co-governance systems in order to determine improvements for such systems.

Please complete the survey as follows:

1. Determine the most important and least important influencing factors.
2. Fill in the codes for the influencing factors.
3. Determine the preference of the most important factor over all the other factors using a number between 1 and 9.
4. Determine the preference of all the other factors over the least important factor using a number between 1 and 9.

Example: In the following tables, A is the most important factor, and C is the least important factor. The preference for A to B is 2, the preference for A to C is 8, and the preference for B to C is 5.

Specify the importance of Correctness and fluidity of public information (*D*_1_), Effectiveness of and engagement in environmental co-governance actions (*D*_2_), and The effect and binding force of environmental governance mechanisms (*D*_3_).

Specify the importance of Atmosphere conducive to the proactive provision of information (*C*_11_), Correct and complete information (*C*_12_), and High-quality information communication platform (*C*_13_).

Specify the importance of Diversified environmental governance mechanisms (*C*_21_), Effective environmental protection projects (*C*_22_), Robust co-management and monitoring mechanisms (*C*_23_), and Smooth joint decision-making by stakeholders (*C*_24_).

Specify the importance of Sufficient funds for environmental governance mechanisms (*C*_31_), Allocated funds (*C*_32_), Penalty for causing environmental damage (*C*_33_), and Satisfactory environmental quality assessment results (*C*_34_).

### Appendix B.2. Modified VIKOR Questionnaire

The purpose of this study is to identify areas for improvement and explore improvement measures by evaluating individuals’ satisfaction with Pingxiang’s environmental co-governance system.

Specify your level of satisfaction with the following factors by using a number between 0 and 10, with 0 indicating “most dissatisfied” and 10 indicating “most satisfied”.

**Table A1:** VIKOR questionnaire.

Criteria	Satisfaction Level (0–10)
Atmosphere conducive to the proactive provision of information (*C*_11_)	
Correct and complete information (*C*_12_)	
High-quality information communication platform (*C*_13_)	
Diversified environmental governance mechanisms (*C*_21_)	
Effective environmental protection projects (*C*_22_)	
Robust co-management and monitoring mechanisms (*C*_23_)	
Smooth joint decision-making by stakeholders (*C*_24_)	
Sufficient funds for environmental governance mechanisms (*C*_31_)	
Allocated funds (*C*_32_)	
Penalty for causing environmental damage (*C*_33_)	
Satisfactory environmental quality assessment results (*C*_34_)	

## Appendix C. Best Worst Method Survey and Calculation Results

The Best Worst Method (BWM) results consist of two parts, namely survey and calculation results.

### Appendix C.1. Survey Results for All Criteria in Each Dimension

In accordance with the BWM operating process, 10 experts were invited to determine the best and worst factors on the basis of their experience and expertise. The survey participants consisted of management personnel from relevant government agencies and scholars of environmental research. Because the survey results for each dimension are presented in the main body of the paper, only the survey results for the criteria are presented in Appendix C.1.

The survey results for the criteria in the dimension of Correctness and fluidity of public information (*D*_1_) are presented in Table A2, Table A3 and Table A4.

**Table A2:** Most and least important factors selected for dimension *D*_1_.

Expert No.	1	2	3	4	5	6	7	8	9	10
Most important	*C*_13_	*C*_13_	*C*_11_	*C*_13_	*C*_13_	*C*_13_	*C*_13_	*C*_11_	*C*_13_	*C*_11_
Least important	*C*_12_	*C*_12_	*C*_12_	*C*_12_	*C*_11_	*C*_12_	*C*_11_	*C*_12_	*C*_11_	*C*_13_

**Table A3:** Best-to-others vector for dimension *D*_1_.

Expert No. 1	*C*_11_	*C*_12_	Expert No. 6	*C*_11_	*C*_12_
*C*_13_	2	3	*C*_13_	1	2
Expert No. 2	*C*_11_	*C*_12_	Expert No. 7	*C*_11_	*C*_12_
*C*_13_	1	3	*C*_13_	2	1
Expert No. 3	*C*_12_	*C*_13_	Expert No. 8	*C*_12_	*C*_13_
*C*_11_	2	2	*C*_11_	3	2
Expert No. 4	*C*_11_	*C*_12_	Expert No. 9	*C*_11_	*C*_12_
*C*_13_	1	1	*C*_13_	2	1
Expert No. 5	*C*_11_	*C*_12_	Expert No. 10	*C*_12_	*C*_13_
*C*_13_	3	1	*C*_11_	2	3

**Table A4:** Others-to-worst vector for dimension *D*_1_.

Expert No. 1	*C*_12_	Expert No. 2	*C*_12_	Expert No. 3	*C*_12_	Expert No. 4	*C*_12_	Expert No. 5	*C*_11_
*C*_11_	2	*C*_11_	2	*C*_11_	2	*C*_11_	1	*C*_12_	2
*C*_13_	3	*C*_13_	3	*C*_13_	1	*C*_13_	1	*C*_13_	3
Expert No. 6	*C*_12_	Expert No. 7	*C*_11_	Expert No. 8	*C*_12_	Expert No. 9	*C*_11_	Expert No. 10	*C*_13_
*C*_11_	2	*C*_12_	2	*C*_11_	3	*C*_12_	2	*C*_11_	3
*C*_13_	2	*C*_13_	2	*C*_13_	2	*C*_13_	2	*C*_12_	2

The survey results for the criteria in the dimension of Effectiveness of and engagement in environmental co-governance actions (*D*_2_) are presented in Table A5, Table A6 and Table A7.

**Table A5:** Most and least important factors selected for dimension *D*_2_.

Expert No.	1	2	3	4	5	6	7	8	9	10
Most important	*C*_21_	*C*_21_	*C*_24_	*C*_21_	*C*_21_	*C*_24_	*C*_21_	*C*_23_	*C*_24_	*C*_23_
Least important	*C*_22_	*C*_22_	*C*_22_	*C*_22_	*C*_22_	*C*_22_	*C*_22_	*C*_22_	*C*_22_	*C*_22_

**Table A6:** Best-to-others vector for dimension *D*_2_.

Expert No. 1	*C*_22_	*C*_23_	*C*_24_	Expert No. 6	*C*_21_	*C*_22_	*C*_23_
*C*_21_	6	4	2	*C*_24_	2	4	2
Expert No. 2	*C*_22_	*C*_23_	*C*_24_	Expert No. 7	*C*_22_	*C*_23_	*C*_24_
*C*_21_	5	3	2	*C*_21_	4	1	2
Expert No. 3	*C*_21_	*C*_22_	*C*_23_	Expert No. 8	*C*_21_	*C*_22_	*C*_24_
*C*_24_	2	4	3	*C*_23_	2	4	3
Expert No. 4	*C*_22_	*C*_23_	*C*_24_	Expert No. 9	*C*_21_	*C*_22_	*C*_23_
*C*_21_	4	3	1	*C*_24_	1	3	2
Expert No. 5	*C*_22_	*C*_23_	*C*_24_	Expert No. 10	*C*_21_	*C*_22_	*C*_24_
*C*_21_	5	2	1	*C*_23_	2	3	1

**Table A7:** Others-to-worst vector for dimension *D*_2_.

Expert No. 1	*C*_22_	Expert No. 2	*C*_22_	Expert No. 3	*C*_22_	Expert No. 4	*C*_22_	Expert No. 5	*C*_22_
*C*_21_	6	*C*_21_	5	*C*_21_	3	*C*_21_	4	*C*_21_	5
*C*_23_	3	*C*_23_	2	*C*_23_	2	*C*_23_	2	*C*_23_	4
*C*_24_	4	*C*_24_	3	*C*_24_	4	*C*_24_	4	*C*_24_	5
Expert No. 6	*C*_22_	Expert No. 7	*C*_22_	Expert No. 8	*C*_22_	Expert No. 9	*C*_22_	Expert No. 10	*C*_22_
*C*_21_	2	*C*_21_	4	*C*_21_	3	*C*_21_	3	*C*_21_	2
*C*_23_	2	*C*_23_	4	*C*_23_	4	*C*_23_	2	*C*_23_	3
*C*_24_	4	*C*_24_	3	*C*_24_	2	*C*_24_	3	*C*_24_	3

The survey results for the criteria in the dimension of The effect and binding force of environmental governance mechanisms (*D*_3_) are presented in Table A8, Table A9 and Table A10.

**Table A8:** Most and least important factors selected for dimension *D*_3_.

Expert No.	1	2	3	4	5	6	7	8	9	10
Most important	*C*_32_	*C*_32_	*C*_32_	*C*_33_	*C*_33_	*C*_32_	*C*_32_	*C*_34_	*C*_32_	*C*_34_
Least important	*C*_31_	*C*_31_	*C*_34_	*C*_34_	*C*_31_	*C*_31_	*C*_31_	*C*_31_	*C*_31_	*C*_31_

**Table A9:** Best-to-others vector for dimension *D*_3_.

Expert No. 1	*C*_31_	*C*_33_	*C*_34_	Expert No. 6	*C*_31_	*C*_33_	*C*_34_
*C*_32_	4	3	1	*C*_32_	6	4	3
Expert No. 2	*C*_31_	*C*_33_	*C*_34_	Expert No. 7	*C*_31_	*C*_33_	*C*_34_
*C*_32_	5	2	4	*C*_32_	3	1	2
Expert No. 3	*C*_31_	*C*_33_	*C*_34_	Expert No. 8	*C*_31_	*C*_32_	*C*_33_
*C*_32_	2	1	3	*C*_34_	4	2	3
Expert No. 4	*C*_31_	*C*_32_	*C*_34_	Expert No. 9	*C*_31_	*C*_33_	*C*_34_
*C*_33_	3	3	4	*C*_32_	3	1	1
Expert No. 5	*C*_31_	*C*_32_	*C*_34_	Expert No. 10	*C*_31_	*C*_32_	*C*_33_
*C*_33_	4	2	3	*C*_34_	3	2	3

**Table A10:** Others-to-worst vector for dimension *D*_3_.

Expert No. 1	*C*_31_	Expert No. 2	*C*_31_	Expert No. 3	*C*_34_	Expert No. 4	*C*_34_	Expert No. 5	*C*_31_
*C*_32_	4	*C*_32_	5	*C*_31_	2	*C*_31_	3	*C*_32_	3
*C*_33_	2	*C*_33_	4	*C*_32_	3	*C*_32_	3	*C*_33_	4
*C*_34_	4	*C*_34_	2	*C*_33_	3	*C*_33_	4	*C*_34_	2
Expert No. 6	*C*_31_	Expert No. 7	*C*_31_	Expert No. 8	*C*_31_	Expert No. 9	*C*_31_	Expert No. 10	*C*_31_
*C*_32_	6	*C*_32_	3	*C*_32_	3	*C*_32_	3	*C*_32_	2
*C*_33_	2	*C*_33_	3	*C*_33_	2	*C*_33_	3	*C*_33_	1
*C*_34_	4	*C*_34_	2	*C*_34_	4	*C*_34_	3	*C*_34_	3

### Appendix C.2. BWM Calculation Results

In accordance with the BWM calculation steps and survey results, we calculated the weight of each factor. First, the weight of each factor was calculated according to the survey results obtained from each expert. Second, the average weight of each factor was calculated. Finally, the CI value was calculated.

Table A11 presents the calculation results for each dimension. Table A12, Table A13 and Table A14 display the calculation results for the criteria. The CI values of all results met the threshold; therefore, the weight values were considered reliable.

**Table A11:** Weights of each dimension.

Dimensions	E1	E2	E3	E4	E5	E6	E7	E8	E9	E10	AVG
Correctness and fluidity of public information (*D*_1_)	0.313	0.385	0.167	0.412	0.313	0.292	0.200	0.400	0.313	0.333	0.313
Effectiveness of and engagement in environmental co-governance actions (*D*_2_)	0.563	0.462	0.600	0.471	0.563	0.542	0.550	0.400	0.563	0.333	0.504
The effect and binding force of environmental governance mechanisms (*D*_3_)	0.125	0.154	0.233	0.118	0.125	0.167	0.250	0.200	0.125	0.333	0.183
$\xi^{L*}$	0.063	0.077	0.1	0.059	0.063	0.042	0.05	0	0.063	0	

**Table A12:** Weights of criteria for dimension *D*_1_.

Criteria	E1	E2	E3	E4	E5	E6	E7	E8	E9	E10	AVG
Atmosphere conducive to the proactive provision of information (*C*_11_)	0.292	0.385	0.500	0.333	0.154	0.400	0.200	0.542	0.200	0.542	0.355
Correct and complete information (*C*_12_)	0.167	0.154	0.250	0.333	0.385	0.200	0.400	0.167	0.400	0.292	0.275
High-quality information communication platform (*C*_13_)	0.542	0.462	0.250	0.333	0.462	0.400	0.400	0.292	0.400	0.167	0.371
$\xi^{L*}$	0.042	0.077	0	0	0.077	0	0	0.042	0	0.042	

**Table A13:** Weights of criteria for dimension *D*_2_.

Criteria	E1	E2	E3	E4	E5	E6	E7	E8	E9	E10	AVG
Diversified environmental governance mechanisms (*C*_21_)	0.500	0.485	0.259	0.386	0.365	0.222	0.360	0.259	0.351	0.189	0.338
Effective environmental protection projects (*C*_22_)	0.071	0.092	0.103	0.088	0.063	0.111	0.080	0.103	0.108	0.108	0.093
Robust co-management and monitoring mechanisms (*C*_23_)	0.143	0.169	0.172	0.140	0.206	0.222	0.360	0.466	0.189	0.351	0.242
Smooth joint decision-making by stakeholders (*C*_24_)	0.286	0.254	0.466	0.386	0.365	0.444	0.200	0.172	0.351	0.351	0.328
$\xi^{L*}$	0.071	0.023	0.052	0.035	0.048	0.000	0.040	0.052	0.027	0.027	

**Table A14:** Weights of criteria for dimension *D*_3_.

Criteria	E1	E2	E3	E4	E5	E6	E7	E8	E9	E10	AVG
Sufficient funds for environmental governance mechanisms (*C*_31_)	0.088	0.086	0.189	0.200	0.103	0.076	0.108	0.103	0.100	0.140	0.119
Allocated funds (*C*_32_)	0.386	0.495	0.351	0.200	0.259	0.550	0.351	0.259	0.300	0.244	0.339
Penalty for causing environmental damage (*C*_33_)	0.140	0.280	0.351	0.500	0.466	0.160	0.351	0.172	0.300	0.163	0.288
Satisfactory environmental quality assessment results (*C*_34_)	0.386	0.140	0.108	0.100	0.172	0.214	0.189	0.466	0.300	0.453	0.253
$\xi^{L*}$	0.035	0.065	0.027	0.100	0.052	0.092	0.027	0.052	0.000	0.035	
